# Nature and Properties of Lateritic Soils Derived from Different Parent Materials in Taiwan

**DOI:** 10.1155/2014/247194

**Published:** 2014-04-27

**Authors:** Tzu-Hsing Ko

**Affiliations:** Department of Tourism Affairs, Tzu Hui Institute of Technology, 367 Sanmin Road, Nanjou Township, Pingtung County, Taiwan

## Abstract

The objective of this study was to investigate the physical, chemical, and mineralogical composition of lateritic soils in order to use these soils as potential commercial products for industrial application in the future. Five lateritic soils derived from various parent materials in Taiwan, including andesite, diluvium, shale stone, basalt, and Pleistocene deposit, were collected from the B_t1_ level of soil samples. Based on the analyses, the Tungwei soil is an alfisol, whereas other lateritic soils are ultisol. Higher pH value of Tungwei is attributed to the large amounts of Ca^2+^ and Mg^2+^. Loupi and Pingchen soils would be the older lateritic soils because of the lower active iron ratio. For the iron minerals, the magnetic iron oxides such as major amounts of magnetite and maghemite were found for Tamshui and Tungwei lateritic soils, respectively. Lepidocrocite was only found in Soka soil and intermediate amounts of goethite were detected for Loupi and Pingchen soils. After Mg-saturated and K-saturated processes, major amounts of mixed layer were observed in Loupi and Soka soils, whereas the montmorillonite was only detected in Tungwei soil. The investigation results revealed that the parent materials would play an important role during soil weathering process and physical, chemical, and mineralogy compositions strongly affect the formation of lateritic soils.

## 1. Introduction

Lateritic soils are one of important soils and are widespread in tropical areas and subtropical climates. They are the most highly weathered soils in the classification system. Lateritic soils in Taiwan are mostly classified into ultisols and alfisols and cover about 25 percent of the cultivation lands. The significant features of the lateritic soils are their unique color, poor fertility, and high clay content and lower cation exchange capacity. In addition, lateritic soils possess a great amount of iron and aluminum oxides [1]. Iron oxides, existing mainly in the amorphous and crystalline inorganic forms, are one of major components in many soil orders. In my previous study, a series of soil samples including alfisol, inceptisol, entisol, and ultisol were used to test their H_2_S removal efficiency from hot coal gas. Experimental results showed that the ultisols have the best removal efficiency among all soil samples. Additionally, the contents of total free iron have been confirmed as the major component to affect overall removal efficiency. It is thus very important to understand the detailed properties of lateritic soils when they are going to be a commercial product for industrial application. Based on the previous study, it is believed that the Tamshui and Tungwei lateritic soils are the best candidates for industrial application because of the presence of magnetite and maghemite, which are two types of iron oxides that have excellent thermodynamic sulfurization compared to other iron oxides [2, 3]. Parent material is a key factor affecting the iron and mineral composition and distribution for lateritic soils. Anda et al. [4] reported a series of oxisols derived from serpentinite, basalt, and andesite and found that the content of iron oxides has an obvious different distribution. Approximately 19% of iron oxide was determined for the lateritic soils derived from serpentinite. Different parent materials also bring the different physical and chemical properties.

Therefore, in order to better understand the detailed information of lateritic soils, the main objectives of this study were to investigate the properties of lateritic soils derived from different parent materials, including shale stone, basalt, diluvium, and andesite, and to provide basic information about lateritic soils for agricultural development.

## 2. Materials and Methods

### 2.1. Study Area

Five lateritic soils used in this study were collected from different locations in Taiwan. They are located at Taipei County (Tamshui), Taoyuan County (Pingchen), Pingtung County (Loupi), Taitung County (Soka), and Penghu County (Tungwei), respectively. A brief description of the morphological characterization for these lateritic soils is given in Table 1. According to the soil classification Tamshui, Pingchen, Loupi, and Soka are ultisol and Tungwei is an alfisol.

### 2.2. Analytical Methods

Soil samples were air-dried, crushed with a mortar, and sieved to remove coarse (>2 mm) fragments. Particle-size distribution was obtained by the pipette method after removal of carbonate, organic matters, and MnO_2_. Carbonate was removed by 1 M NaOAc with pH = 5 at 60°C and organic matters and MnO_2_ were digested by 30% H_2_O_2_ [5]. Soil pH value was measured on a mixture of 1 : 1 soil/deionized water and 1 : 1 soil/1 M KCl solution by glass electrode, respectively [6]. Organic matter content was determined by the Walkley-Black wet oxidation method [7]. Cation exchange capacity was determined by the ammonium acetate method at pH = 7 [8]. Free Fe (Fe_d_) was extracted by the dithionite-citrate-bicarbonate (DCB) method [9]. Acid ammonium oxalate in the dark was used to extract noncrystalline (poorly crystalline and organically bound) Fe (Fe_ox_) [10]. The concentration of exchangeable cations and Fe was determined by ICP/AES (JY38P model, JOBIN YVON). Mineralogical composition was detected by X-ray powder diffraction for the clay specimens. The clay specimens were saturated with 0.5 M MgCl_2_ (Mg-saturated) and 1 M KCl (K-saturated), respectively. The expansion properties of the Mg-saturated clay specimens were determined using ethylene glycol solvation at 65°C for 24 hours. The K-saturated clay specimens were subjected to successive heat treatment at 110, 350, and 550°C for 2 hours. The oriented clay specimens were examined with a Rigaku Model D/MAX III-V X-ray powder diffractometer equipped with a Ni-filtered CuK*α* radiation generated at 30 mA and 40 kV. The diffraction patterns were recorded from 3° to 90° with a scan rate of 3°/min. The identification and semiquantitative determination of the clay minerals were based on the difference of reflection patterns from the K-saturated, Mg-saturated, glycolated, heated, and air-dried specimens [11, 12].

## 3. Results and Discussion

### 3.1. Basic Physical and Chemical Properties of Various Lateritic Soils

Brief descriptions of some physical and chemical properties as well as parent conditions of the collected soils are shown in Tables 2 and 3. Munsell soil color notation of these soils appears in 2.5 to 5YR, indicating that the color of these soils is red or reddish brown. Loupi, Soka, and Tungwei contain high amounts of clay fraction, while Tamshui and Pingchen consist mostly of silt fraction. They belong to clay and silty clay in texture classification, respectively. Except for Pingchen, all the soils possess moderate structure. Tamshui and Tungwei appear moderate and very fine granular in structure; the others appear subangular blocky and angular blocky. The pH (H_2_O) values of the soils are 4.85, 4.06, 4.02, 4.46, and 8.13 for Tamshui, Pingchen, Loupi, Soka, and Tungwei, respectively. Obviously, all soils are acidic in nature except Tungwei. The difference in pH (pH_KCl_-pH_H_2_O_) shows a negative value for all soils, suggesting that the dominative charge on the surface of all soils is negative. On the other hand, this also indicates that a portion of the exchange sites have hydrogen ions. This provides anion exchange capacity and lessens the value for cation exchange capacity. At pH 7, the hydrogen ions are gone, and thus the cation exchange capacity is an inflated value. In the case of Tungwei, its pH value belongs to alkaline region. This is due to the fact that this site contains large amounts of calcium carbonate and shell nodules. Therefore, the exchangeable cations of Ca^2+^ and Mg^2+^ for Tungwei are 9.28 and 8.73 (cmol kg^−1^), respectively. The value is significantly higher than that of other lateritic soils, indicating that the high pH value for Tungwei resulted from great amounts of Ca^2+^ and Mg^2+^.

The free iron oxides or DCB extractable iron oxides (Fe_d_) in five soils being studied range from 3.86 to 13.8%. The oxalate extractable iron oxides (Fe_ox_) contents of the five soils are very low. Values of Fe_ox_ in five soils range from 0.36 to 2.42%. This result reflects that iron oxides in lateritic soils contain less amounts of poor crystalline or amorphous form of iron oxides and the major form of iron oxides present in soil is crystalline iron oxides. The ratio of Fe_ox_ to Fe_d_ has been expressed as the “active iron ratio” by Lekwa and Whiteside [13]. In this study, the active iron ratio for Loupi and Pingchen is less than that of Tamshui and Tungwei. This result can provide the evidence regarding soil-forming ages [14, 15]. The Fe_ox_ to Fe_d_ ratio of the five lateritic soils follow the order Tungwei > Tamshui > Soka > Pingchen > Loupi. This implies that Loupi may be the oldest lateritic soils compared to others.

### 3.2. Clay Mineralogy of Lateritic Soils

The mineralogical composition for the five lateritic soils is tabulated in Table 4. The major difference among these soils is the content of iron oxides. The dominant iron species are magnetite and maghemite for the Tamshui and Tungwei. These two soil samples possess the magnetic iron species probably due to their parent material conditions. Parent materials of Tamshui and Tungwei are andesite and basalt, respectively, which belong to igneous rock. Due to younger parent materials or landscapes, the extent of weathering or chemical leaching is less intensive and the presence of magnetite and maghemite is ascribed to this reason. Unlike Tamshui and Tungwei, Pingchen and Loupi contain identical iron oxides species (goethite and less hematite) and the main iron oxide species contained in Soka is lepidocrocite. In general, the hematite is the stable phase for iron oxides in the atmosphere. Taiwan is located at the boundary between tropical and subtropical climate. The average annual precipitation is about 2,400 mm and average temperature is about 23°C. Under such high humidity condition, hematite is transformed into goethite or lepidocrocite. For all five soils, minor amounts of hematite are detected by XRD. After K- and Mg-saturated treatment, some clay minerals are also identified in this study. Pingchen and Loupi possess the same clay minerals including kaolinite, micas, gibbsite, vermiculite, and minor mixed layer chlorite. Soka contains large amounts of quartz, micas, and mixed layer clay minerals along with small amounts of chlorite and gibbsite. Uniquely, the clay minerals in Tamshui and Tungwei are not conspicuousness. Only montmorillonite is detected in Tungwei soil. On the basis of the chemical and mineralogy analysis, it can be ascertained that the difference among lateritic soils in Taiwan resulted from difference among various parent materials. The parent materials play an important factor in soil-forming process for lateritic soils. The extent of weathering probably decreases in the order Loupi *≒* Pingchen > Soka > Tamshui > Tungwei.

## 4. Conclusions

In this study five lateritic soils formed from various parent materials in Taiwan were examined to understand their physical, chemical, and mineralogy properties. Results revealed that the parent materials play an important role during soil weathering. The physical, chemical, and mineralogy compositions strongly affect the formation of soil. The Pingchen and Loupi lateritic soils likely have stronger weathering process, whereas the Tungwei has the younger soil-forming age. The most difference among all lateritic soils is their content of iron oxides. The Tamshui and Tungwei lateritic soils were found to have magnetic iron oxides. Magnetite and maghemite are the major iron oxide for Tamshui and Tungwei, respectively. Lepidocrocite was only found in Soka lateritic soils and intermediate amount of goethite was determined for Loupi soils.

## Figures and Tables

**Table 1:** Morphological characterization of the lateritic soils being studied.

Sample locations	Parent materials	Soil family and great soil groups
Tamshui	Andesite	Very fine, mixed, hyperthermic, and typic paleudult
Pingchen	Pleistocene deposit	Fine, mixed, hyperthermic, and rhodic paleudult
Loupi	Diluvium	Fine-loam, mixed, hyperthermic, and typic paleudult
Soka	Shale stone	Fine-loam, mixed, hyperthermic, and typic hapludult
Tungwei	Basalt	Fine, mixed, hyperthermic, and typic rhodustalf

**Table 2:** Some physical properties of the lateritic soils being studied.

Sample	Depth (cm)	Horizon	Munsell color (dry)	Sand	Silt	Clay	Texture	Structure^a^	Consistence
				(%)			
Tamshui	0–10	A	2.5YR 3/4	11.5	45.6	42.9	Silty clay	2-vf-gr	Very friable
Pingchen	0–10	Ap1	5YR 6/8	14.4	43.8	41.8	Silty clay	1-vf-sbk	Hard
Loupi	0–10	Ap1	5YR 5/6	14.3	34.2	51.5	Clay	2-f-sbk	Friable
Soka	0–10	A	5YR 4/5	23.5	26.8	49.7	Clay	2-f-abk	Firm
Tungwei	0–10	A	2.5YR 3/4	17.7	22.1	60.2	Clay	2-vf-gr	Firm

^a^1: weak; 2: moderate; vf: very fine; f: fine; gr: granular; sbk: subangular blocky; abk: angular blocky.

**Table 3:** Some chemical properties of the lateritic soils being studied.

Sample	pH H_2_O	pH KCl	ΔpH KCl − H_2_O	CEC* (cmol/kg)	Organic matters (g/kg)	BSP (%)	Fe_d_ (%)	Fe_ox_ (%)	Fe_ox_/Fe_d_
Tamshui	4.85	4.03	−0.82	12.3	15.8	17.4	6.75	1.06	15.7
Pingchen	4.06	2.94	−1.12	8.9	23.1	7.23	3.86	0.36	9.32
Loupi	4.02	3.39	−0.63	8.4	32.4	3.12	5.31	0.47	8.85
Soka	4.46	3.74	−0.72	13.8	3.5	87.3	8.74	1.03	11.8
Tungwei	8.13	7.31	−0.82	18.7	26.5	23.8	13.8	2.42	17.6

*CEC values are for pH 7.

**Table 4:** Minerals composition in the clay fraction for the five lateritic soils being studied.

Soils location	Mineralogical composition												
	Qz^a^	Kao	Mic	Gib	Hem	Goe	Lep	Mag^b,c^	Maghem^b,c^	Ver	Mon	Chl	ML
Tamshui	++	+	++	+	+	+	nd	+++	++	nd	nd	+	+
Pingchen	++++	++	+++	++	+	++	nd	nd	nd	++	nd	+	++
Loupi	++++	++	+++	++	+	++	nd	nd	nd	++	nd	+	+++
Soka	+++	+	+++	++	+	+	++	nd	nd	nd	nd	++	+++
Tungwei	++	+	+	+	+	+	nd	+	+++	+	+++	nd	+

^a^Qz: quartz or halloysite; Kao: kaolinite; Mic: micas; Gib: gibbsite; Hem: hematite; Goe: goethite; Lep: lepidocrocite; Mag: magnetite; Maghem: maghemite; Ver: vermiculite; Mon: montmorillonite; Chl: chlorite; ML: mixed layer.

^b^Clay fraction without removing free iron oxides.

^c^Magnetite and Maghemite were concentrated by hand magnet.

++++: dominant; +++: major; ++: intermediate; +: minor; nd: not detected.

## References

[1] Shaw JN (2001). Iron and aluminum oxide characterization for highly-weathered Alabama ultisols. *Communications in Soil Science and Plant Analysis*.

[2] Ko T-H (2008). Removal of hydrogen sulfur from coal-derived gas by iron oxides in various oxisols. *Environmental Engineering Science*.

[3] Ko T-H, Chu H, Lin H-P, Peng C-Y (2006). Red soil as a regenerable sorbent for high temperature removal of hydrogen sulfide from coal gas. *Journal of Hazardous Materials*.

[4] Anda M, Shamshuddin J, Fauziah CI, Omar SRS (2008). Mineralogy and factors controlling charge development of three oxisols developed from different parent materials. *Geoderma*.

[5] Gee GE, Bauder JW, Klute A (1986). Particle-size analysis. *Methods of Soil Analysis*.

[6] McLean EO, Page AL, Miller RH, Keeney DR (1982). Soil pH and lime requirement. *Methods of Soil Analysis*.

[7] Nelson DW, Sommer LE, Page AL, Miller RH, Keeney DR (1982). Total carbon, organic carbon, and organic matter. *Methods of Soil Analysis*.

[8] Rhoades JD, Page AL, Miller RH, Keeney DR (1982). Cation exchange capacity. *Methods of Soil Analysis*.

[9] Mehra OP, Jackson ML Iron oxides removed from soils and clays by a dithionite citrate system buffered with sodium bicarbonate.

[10] Schwertmann U (1964). Differenzierung der eisenoxide des bodens durch extraktion mit ammoniumoxalat-loesung. *Zeitschrift für Pflanzenernährung, Düngung, Bodenkunde*.

[11] Brindley GW (1980). Quantitative X-ray mineral analysis of clays. *Crystal Structures of Clay Minerals and Their X-Ray Identification*.

[12] Brown G, Brindley GW (1980). X-ray diffraction procedures for clay mineral identification. *Crystal Structures of Clay Minerals and Their X-Ray Identification*.

[13] Lekwa G, Whiteside EP (1986). Coastal plain soils of southeastern Nigeria: 6 forms of extractable iron, aluminum, and phosphorus. *Soil Science Society of America Journal*.

[14] Schwertmann U (1985). The effect of pedogenic environments on iron oxide minerals. *Advances in Soil Science*.

[15] Aniku JRF, Singer MJ (1990). Pedogenic iron oxide trends in a marine terrace chronosequence. *Soil Science Society of America Journal*.

